# Protein quality control: from mechanism to disease: EMBO Workshop, Costa de la Calma (Mallorca), Spain, April 28 – May 03, 2019

**DOI:** 10.1007/s12192-019-01040-9

**Published:** 2019-11-11

**Authors:** Harm H. Kampinga, Matthias P. Mayer, Axel Mogk

**Affiliations:** 1 Department of Biomedical Science of Cells & Systems, University Medical Center Groningen, University of Groningen, Groningen, The Netherlands; 2 Center for Molecular Biology (ZMBH), University of Heidelberg, Im Neuenheimer Feld 282, 69120 Heidelberg, Germany

**Keywords:** Protein quality control, Protein homeostasis, Chaperones, Protein folding disease

## Abstract

The cellular protein quality control machinery with its central constituents of chaperones and proteases is vital to maintain protein homeostasis under physiological conditions and to protect against acute stress conditions. Imbalances in protein homeostasis also are keys to a plethora of genetic and acquired, often age-related, diseases as well as aging in general. At the EMBO Workshop, speakers covered all major aspects of cellular protein quality control, from basic mechanisms at the molecular, cellular, and organismal level to medical translation. In this report, the highlights of the meeting will be summarized.

## Introduction

The EMBO workshop was held at the Marítim Galatzó Hotel in Costa de la Calma on the wonderful island Mallorca, Spain, and excellently organized by Bernd Bukau (chair) and Eilika Weber-Ban (co-chair). The bi-annual, EMBO-sponsored meeting on protein quality control was, among others, generously co-sponsored by the Cell Stress Society International (CSSI). There were 217 registrants from 40 countries and the program included 35 invited speakers and one keynote lecture by Anne Bertolotti, 31 short talks selected from abstracts as well as flash talks, and 145 poster presentations. In addition, Round Table Lunches and ample time for informal discussions and networking as well as a number of social activities made this meeting a great and successful event. In this report, we have summarized what was presented in the various oral presentations. Each section was sent to the respective speakers mentioned in that section for approval and feedback. While we tried to respond to their comments, we would like to stress that the content of the report and the selection of (limited) citations is fully the responsibility of the authors.

## Chaperone mechanisms

The meeting was opened by Jim Bardwell who presented his work on the ATP-independent chaperones Spy/Hde that assist protein folding in the periplasm of *E. coli* (Horowitz et al. 2018). These chaperones bind clients through a process driven by electrostatic, not hydrophobic, interactions (Koldewey et al. 2016). By these (dynamic) interactions, hydrophobic sequences in the substrate proteins are insulated and this helps the substrate to slowly obtain its final fold. The implications are of particular relevance in light of the fact that the environment *E. coli* faces often is highly acidic (like in the stomach) and contains high salt concentrations, conditions under which proteins readily aggregate and hence require chaperones that can work under such conditions (Stull et al. 2018).

Ulrich Hartl reported on Hsp70-assisted folding of the multi-domain model protein firefly luciferase. At concentrations below 1 nM, luciferase folds spontaneously very slowly and inefficiently, whereas in the presence of the *E. coli*, Hsp70 system DnaK, DnaJ, and GrpE luciferase refolding is efficient and about 20-fold faster. N-terminal and C-terminal domains of luciferase both fold efficiently when separated but misfold in the full-length protein, as shown using single-molecule Förster resonance energy transfer (FRET). DnaK together with DnaJ unfolded misfolded luciferase to a degree that is close to the denatured state, consistent with earlier observations on luciferase and rhodanese (Kellner et al. 2014; Sharma et al. 2010). GrpE-mediated nucleotide exchange leads to release of luciferase, allowing a fraction of molecules to fold rapidly to the native state in every binding and release cycle.

To better understand the cooperation of Hsp70s with their J-domain co-chaperones, Rina Rosenzweig investigated the interaction of the human class A and class B J-domain proteins DnaJA2 and DnaJB1 with human Hsp70 using NMR spectroscopy. Both DnaJA2 and DnaJB1 are considered general-purpose J-domain proteins targeting many diverse protein substrates to Hsp70s, in particular misfolded proteins. They are structurally very similar, containing the N-terminal J-domain (JD), a Gly-Phe-rich (G/F) region, two homologous β-sandwich domains, and a C-terminal dimerization domain (Kampinga and Craig 2010). Where the two proteins differ is that DnaJA2, like all class A J-domain proteins, contains in addition a zinc-finger inserted into the first β-sandwich domain, whereas DnaJB1 has a slightly longer G/F-region. The Rosenzweig group found that the interaction surface of a JD-GF construct of DnaJA2 bound to Hsp70 was very similar to the interaction surface of the JD of *E. coli* DnaJ bound to *E. coli* DnaK·ATP in a recent crystal structure (Kityk et al. 2018), with the GF region having no influence on the interaction. In contrast, a JD-GF construct of DnaJB1 did not interact at all with Hsp70, whereas the JD alone did. Structural investigation revealed that the GF region of DnaJB1 was not completely unstructured, as previously expected, but rather contained a small helix that bound to the JD, preventing interaction with Hsp70. This inhibition was released in the full-length protein, but only when Hsp70 contained its C-terminal EEVD motif. This then provides a structural explanation for the earlier observation that the EEVD motif is essential for chaperone function of yeast Hsp70 in combination with the class B J-domain protein Sis1, but not in combination with the class A J-domain protein Ydj1 (Yu et al. 2015), which does not contain the G/F inhibition of the JD. The EEVD motif interacted with the first β-sandwich domain, consistent with an earlier crystal structure (Li et al. 2006), allosterically releasing the G/F helix from the JD and allowing interaction of the JD with Hsp70. This mechanism is essential for DnaJB1/Hsp70 chaperone activity. Why this interaction type evolved for class B but apparently not for class A J-domain proteins remains to be explored.

The JD-Hsp70 interaction was also the main focus of Jaroslaw Marszalek who reported on an evolutionary approach with docking and molecular dynamic simulations. The focus of the reported investigation was the intriguing case of the independent evolution of specific JD-Hsp70 pairs for chaperoning the assembly factor for the synthesis and transfer of FeS-clusters. A specific J-domain protein exists in all organisms for this chaperoning task, but the Hsp70 partner is either a specialized Hsp70 like the bacterial HscA and the yeast mitochondrial Ssq1 or the general mitochondrial Hsp70 as in human mitochondria. The *K*_M_ values for the JD-Hsp70 interaction are orders of magnitude lower for the specialized JD-Hsp70 pairs than if a general Hsp70 is the partner protein. The JD is responsible for the increased affinity since the interacting residues are more variable than the interaction interface in Hsp70s. These results suggest that the JD-Hsp70 interaction is both evolvable and tuneable.

The thermodynamics of chaperone action was the focus of Paolo De Los Rios’ contribution. In an earlier publication, he and his collaborators had demonstrated that both the GroEL-GroES machine and the Hsp70 system use the energy of ATP to shift proteins (MDH and Luciferase, respectively) out of thermodynamic equilibrium, to keep them in an active state under conditions in which the thermodynamic equilibrium favors the denatured state (Goloubinoff et al. 2018). There they proposed an unfolding model mechanism of action of these chaperones inspired by the Iterative Annealing Model (Todd et al. 1996). In a more recent work, De Los Rios and collaborators have provided a molecular picture for the conversion of the ATP energy into the expansion of the bound substrate by Hsp70 (Assenza et al. 2019). They have shown that the ATPase-driven collective binding of Hsp70s leads to a level of substrate expansion higher than would be possible at thermodynamic equilibrium, which would then naturally correspond, upon substrate release, to a native state population higher than would be possible at equilibrium.

A new co-chaperone for the endoplasmic reticulum Hsp70 BiP was introduced by David Ron. Mesencephalic astrocyte–derived neurotrophic factor (MANF) interacts with BiP in the apo or ADP bound state and stabilizes this state. A crystal structure of MANF in complex with the nucleotide-binding domain of BiP suggests that MANF binding is incompatible with the ATP bound open conformation. MANF inhibits nucleotide exchange factor–stimulated release of ADP and ATP binding. Consequently, MANF inhibits ATP-stimulated substrate release. This function of MANF is reminiscent of the function of HIP for cytosolic Hsp70 (Li et al. 2013); however, MANF binds to a different face of the nucleotide-binding domain.

The interplay of the Hsp70 and Hsp90 chaperone machineries was the topic of the presentation by Johannes Buchner. To identify new modulators of the activity of Hsp90 clients, they used a CRISPRi screen in combination with a steroid hormone receptor–dependent reporter. They identified NudC as a potential novel co-chaperone. NudC contains a C-terminal CS-domain found also in other Hsp90 co-chaperones like p23 and Sgt1. It seems to affect both the Hsp70 and Hsp90 system and thus may affect the coordination of the Hsp70 and Hsp90 machineries.

A Hop-independent interaction of the Hsp70 and Hsp90 chaperones was reported by Didier Picard. Hsp70 and Hsp90 are both essential in all eukaryotic cells; however, deletion of Hop/Sti1 produces a rather mild phenotype. Deletion of Sti1 in yeast caused temperature sensitivity and knock-out of Hop in human cells had no effect on proliferation. The whole cell proteome barely changed and levels of Hsp90 client proteins were not affected, except for glucocorticoid receptor. Surprisingly, Hop^−/−^ cells are more resistant to a 43 °C heat shock and to proteasome inhibitors. Luciferase refolds more efficiently in Hop^−/−^ cells and polyQ aggregation is reduced, indicating that the cellular chaperone capacity is increased. In contrast, the proteasome activity is reduced in Hop^−/−^cells, but not the levels of the proteasomal subunits. The assembly of the 30S particle seems to be affected in the absence of Hop, indicating that the Hsp70-Hsp90 machinery in conjunction with Hop is necessary for proteasome assembly. The data suggest that some clients depend on the Hop-mediated interaction of the Hsp70 and Hsp90 machine, whereas other clients use the more ancient pathway found in bacteria in which Hsp70 directly interacts with Hsp90 without Hop. The presumed interaction surface seems to be conserved as certain amino acid replacements in Hsp90 abrogate co-precipitation of Hsp90 with Hsp70.

Judith Frydman reported on her work with TRiC, the mammalian cytosolic type II chaperonin. TRiC is composed of two rings of eight different subunits. It was shown how the different TRiC/CCT subunits create an asymmetry crucial for its ATP-driven conformational cycle and a charge distribution inside the chamber for its interaction with substrates. Next, she showed how TRiC cooperates with the chaperone prefoldin/GIMc (PFD). In contrast to what is generally assumed, prefoldin does not bind substrates first and “presents” these to TRiC. Rather, PFD enhances the rate and yield of folding of substrates bound to TRiC, via binding to TRiC through a conserved interface of electrostatic contacts. Abolishing the TRiC-PFD interaction in vivo led to the accumulation of toxic amyloid aggregates (Gestaut et al. 2019).

## Functions, structures, and mechanisms of AAA+ proteins

A couple of presentations reported on recent structural and mechanistic findings on AAA+ proteins. AAA+ proteins form ring-like hexamers, which unfold or dissociate substrate proteins and protein assemblies under ATP consumption. This activity relies on aromatic residues that are located at the pore site of an AAA+ hexamer and thread substrates through the central channel. Recent cryo electron microscopy (cryoEM) structures revealed that AAA+ proteins form asymmetric hexamers with the ATPase subunit existing in diverse activity states. A sequential change in the subunit activity states is suggested to drive a rotary threading mechanism (de la Pena et al. 2018; Gates et al. 2017).

Daniel Southworth reported on cryoEM structures of the bacterial disaggregase ClpB, which is composed of two ATPase domains (AAA1, AAA2) that form separate, staggered ATPase rings. ClpB is repressed by regulatory coiled-coil M-domains, allowing for controlled ClpB activation by an Hsp70 partner chaperone (Mogk et al. 2018). Southworth showed structures of the activated ClpB-K476C M-domain mutant in complex with a casein substrate. The high-resolution (2.9 Å) structure allowed a very detailed characterization of the nature of substrate contact sites within the ClpB channel in great detail (Rizo et al. 2019). Structural analysis and computational modeling revealed that ClpB preferentially interacts with aromatic and hydrophobic side chains, providing specificity for unfolded proteins. The comparison of two distinct ClpB-K476C cryoEM structures revealed that inactivation of an AAA1 subunit is directly coupled to substrate release and pore loop repositioning. This further supports a sequential and stepwise threading mechanism driving protein disaggregation. Helen Saibil reported on additional, distinct cryoEM structures of the same activated ClpB mutant with substrate bound. They indicate that ClpB activation triggers a sequential ATPase and threading mechanism in the AAA2 ring, which represents the main threading motor. AAA1 and AAA2 rings of activated ClpB do not seem to work synchronously but in alternating cycles, suggesting that ClpB threads substrates via a rope-climbing mechanism (Deville et al. 2019).

Kursad Turgay reported on *Bacillus subtilis* ClpC, which represents a central AAA+ protein in the proteostasis network of this Gram-positive model organism. ClpC cooperates with diverse adapter proteins, which target specific substrates and concurrently stimulate ClpC ATPase activity (Kirstein et al. 2009). He showed that in *B. subtilis* cells, the McsB adapter protein is necessary for ClpC-mediated disaggregation. In vitro reconstitution revealed that ClpC/McsB works in conjunction with McsA and YwlE with similar efficiency as the canonical bacterial ClpB/Hsp70 disaggregation system. Interestingly, McsB acts as adaptor protein with a concurrent protein arginine kinase activity targeting and phosphorylating substrate proteins. This activity is induced by McsA and counterbalanced by the phosphatase YwlE (Elsholz et al. 2012; Fuhrmann et al. 2009).

Tom Rapoport reported on the cryoEM structure of the yeast AAA+ dislocase Cdc48 in complex with substrate and the Ufd1/Npl4 adapter protein complex. Cdc48 is essential for the extraction of misfolded protein from the ER for proteasomal degradation in the cytosol (ERAD) (Wu and Rapoport 2018). The Cdc48 function in ERAD requires cooperation with the Ufd1/Npl4 partner, which targets poly-ubiquitinated proteins to Cdc48. The structure of the complex revealed substrate binding inside the Cdc48 channel and the interaction of an unfolded Ubiquitin moiety with Npl4. This suggests that the poly-Ubiquitin chain is sequentially unfolded by Cdc48 and co-threaded along with the substrate protein (Twomey et al. 2019). Rapid refolding of Ubiquitin after completion of threading is suggested to supersede the need for substrate re-ubiquitination and ensuring direct targeting to the proteasome.

Hemmo Meyer showed that the human ortholog of Cdc48, p97/VCP, also functions as a Ubiquitin-independent protein complex disassembling enzyme and that this is mediated by an alternative adapter and is relevant for protein phosphatase PP1 maturation. PP1 acts on a multiplicity of cellular targets. Its specificity is determined by partner proteins that function as substrate specifiers. The formation of the distinct mature PP1 complexes is preceded by binding of SDS22 and I3 to newly synthesized PP1. This interaction keeps PP1 inactive and dissociation of SDS22/I3 is therefore required to allow for PP1 partner binding and the formation of functional holoenzymes. Meyer demonstrated that the p97-adapter protein p37 binds via its SEP domain to I3. This allows for recruitment of p97/VCP, which unfolds and threads I3 through its central channel under ATP consumption, thus stripping I3 from PP1 and disassembling the entire PP1 complex (Weith et al. 2018).

Irina Gutsche presented new structural findings on the MoxR AAA+ protein RavA, which interacts with the Lysine decarboxylase LdcI. LdcI protects *E. coli* cells from acid stress by buffering pH. Five RavA hexamers bind to two pentameric LdcI disks forming a cage-like structure (Malet et al. 2014). RavA interaction is mediated by LARA domains that are connected via a helical bundle domain to the hexameric ATPase ring. New cryoEM data on the structure of the LdcI-RavA cage reveals spiral organization of RavA, with the seams constrained to two opposing orientations. In parallel, cryoEM structures of RavA alone show that it exists as a mixture of a spiral and a planar C2-symmetric state. This suggests a mechanism of RavA ATPase activity in which ATP hydrolysis does not progress sequentially around the RavA spiral, but instead switches between opposing positions across the hexamer.

Matthew Wohlever reported on the mitochondrial AAA+ protein Msp1 that acts in the sorting control of tail-anchored (TA) proteins. TA proteins harbor a single C-terminal transmembrane domain for integration into the ER membrane or the outer membrane of mitochondria (OMM). TA proteins that mislocalize to the OMM are recognized and removed by Msp1 (Okreglak and Walter 2014). Wohlever reported on the reconstitution of Msp1 activity in liposomes, demonstrating that it is sufficient for the extraction of mistargeted TA proteins (Wohlever et al. 2017). The in vitro system now allows for dissection of how Msp1 distinguishes falsely from correctly inserted TA proteins and the role of lipid composition in the recognition process. Initial data point to binding of Msp1 to exposed hydrophobic residues of a TA model substrate.

## Co-translational protein folding and complex assembly and guidance by ribosome-associated chaperones

The folding of nascent polypeptides and their subsequent assembly with partner proteins occurs in the densely crowded cellular environment. Recent findings demonstrate that the engagement of interacting protein subunits and thus the formation of protein assemblies can happen co-translationally and is prevalent in eukaryotes (Shiber et al. 2018).

Martine Collart reported on the co-translational assembly of the proteasome components Rpt1 and Rpt2. Rpt1-Rpt2 interaction involves ribosome pausing, allowing for prolonged exposure of interaction sites. Rpt1 and Rpt2 translation is continued upon partner association (Panasenko et al. 2019). The co-localization of Rpt1/2-encoding mRNAs might facilitate co-translational interactions. The mRNAs locate to specific structures, assemblysomes, which might include additional factors for proteasome assembly. Not1, the scaffold of the Ccr4-Not complex, is a component of assemblysomes and mediates col-localization of *RPT1* and *RPT2* mRNAs.

Matilde Bertolini (Bernd Bukau Lab) showed that homo-oligomers can form co-translationally by interaction of two nascent subunits. This “co-co-assembly” mechanism is detected by disome-selective ribosome profiling and is prevalent in human cells. The interactions frequently involve coiled-coil dimerization motifs as co-translational assembly sites. The interacting nascent chains likely arise from the same mRNA, pointing to a cis-assembly process. This could ensure the formation of isoform-specific dimers and prevent heterodimerization of spliced isoforms.

Jonathan Weissman reported on the identification of novel, small (< 100 amino acids) open reading frames (ORFs) in the human genome. Those were identified by ribosome profiling in the presence of the drug Harringtonin, which slows translation initiation leading to a pile-up of ribosomes at initiation sites. Identified ORFs use CUG as start codon and are synthesized via non-canonical translation. They frequently interact with downstream-encoded proteins and are part of respective protein complexes. This points towards the presence of “functional operons” in mammalian cells. The small ORFs might guide folding and complex formation of “downstream” proteins or control their translation.

Ribosome-associated chaperones co-translationally interact with nascent polypeptide chains to support folding or to guide them to the correct cellular compartment (Kramer et al. 2018). Irmgard Sinning reported on the structure and mechanism of the RAC complex, composed of the ribosome-associated J-protein Zuo1 and the non-canonical Hsp70 Ssz1 (Zhang et al. 2017). Zuo1 interacts with Ssz1 and completes the Ssz1 substrate-binding domain (SBD). This interaction involves a proline-rich motif that binds as a pseudo-substrate and stabilizes the Ssz1 SBD. This motif can compete with nascent polypeptides for binding to Ssz1, thereby potentially regulating translation.

Sabine Rospert continued on the role of RAC and the ribosome-associated Hsp70 Ssb1/2 in controlling translation fidelity in yeast cells. The chaperones control the translation process in two ways (Gribling-Burrer et al. 2019). First, Zuo1 suppresses translational pausing at internal stalling-prone sequences (e.g., poly(A)-like sequences). Second, RAC and Ssb1/2 play a role in ribosome biogenesis and loss of RAC or Ssb1/2 function leads to assembly of ribosomes with structural changes in the decoding center. Addition of purified RAC to ribosomes isolated from ∆zuo1 cells rescues the stalling defect on polylysine encoding sequences but not the read-through at stop codons, indicating that the defects in the decoding center cannot be mended after the assembly of the ribosomal subunits. These ribosomes are impaired in discriminating sense codons from stop codons and become sensitive to the antibiotic paromomycin, which does not bind to eukaryotic wild-type ribosomes, but it binds to ribosomes assembled in cells lacking the RAC system.

Elke Deuerling reported on the ribosome-associated NAC complex composed of αNAC and βNAC (Deuerling et al. 2019). She showed that the N-terminal extension (NTE) of αNAC harbors a conserved “DSD” motif, which negatively regulates NAC ribosome binding. The cryoEM structure of NAC bound to a vacant ribosome revealed that the NAC binding site overlaps with SRP and RAC. Remarkably, the NTE of βNAC enters the ribosomal exit tunnel until the constriction site, thus functioning as a plug (Gamerdinger et al. 2019). This might prevent interactions of vacant ribosomes with SRP or ER translocons, rationalizing mistargeting of proteins to the ER in *C. elegans* upon NAC depletion (Gamerdinger et al. 2015). Upon translation start, the growing nascent polypeptide chain pushes the βNAC NTE out of the exit tunnel.

Pierre Genevaux introduced bacterial antitoxins, which are dependent on a specific SecB-like chaperone for folding (Bordes et al. 2016). Toxin-antitoxin systems are widespread in bacteria and arrest cell growth in response to specific adverse growth conditions by antitoxin inactivation. Genevaux showed that the *M. tuberculosis* (Mtb) HigA antitoxin harbors a C-terminal chaperone addiction sequence (ChAD) that hampers antitoxin folding and makes the folding process dependent on a specialized SecB chaperone. This might couple antitoxin stability to stress conditions depleting SecB function. The crystal structure of a ChAD peptide in complex with Mtb SecB revealed a specific ChAD-binding site not employed by SecA or secretory proteins (Guillet et al. 2019).

## Cellular quality control of stalled ribosomes

Specific mRNA sequences and respective stretches of nascent polypeptide chains modulate translation speed and can even cause translational pausing. Hideki Taguchi reported on “intrinsic ribosome destabilization sequences” (IRD) on mRNAs in *E. coli*, which destabilize ribosomes and even cause ribosome dissociation from mRNA (Chadani et al. 2017). IRDs include a consecutive array of acidic residues, interrupted by prolines. Taguchi reported on the identification of natural IRDs found in the MgtL leader peptide of the MgtA Mg^2+^ transporter. Also, IRD plays a role in bypassing an internal untranslated region of the gp60 mRNA, producing a T4 phage topoisomerase. IRDs might fulfill multiple functions including prophage excision, regulation of protein translation, and expansion of the genetic repertoire by allowing for formation of truncated or full-length proteins from the same mRNA.

Cleaved, truncated mRNA lacking a stop codon or stop codon read-through causes ribosome stalling and the exposure of incomplete or erroneous proteins. Stalled ribosomes are specifically recognized and recycled by a cellular surveillance system, whereas the aberrant polypeptide chains are instantly marked for degradation. Ramanujan Hegde presented the cryoEM structure of a ribosome stalled during translation of a poly(A) tail of an mRNA. He showed that the poly(A) sequence inside the ribosome’s mRNA channel forms a helix that is capped on both sites by rRNA bases. This mRNA-rRNA conformation prevents tRNA binding to the ribosomal A-site. Additionally, he showed that the presence of polylysine [encoded by poly(A)] in the ribosome exit tunnel slows down peptide bond formation, providing sufficient time for mRNA/rRNA stacking. He concluded that this coincidence detection mechanism enables ribosomes to identify mRNAs that have been inappropriately polyadenylated within the coding region.

Toshifumi Inada reported on endogenous mRNA/protein sequences that induce translation stalling and subsequent RQC recruitment. Sequential ubiquitination of non-functional ribosomes is crucial for subunit dissociation and is mediated by monoubiquitination (Mag2) and polyubiquitination (Hel2 and Rqt2) of uS3. The sequential uS3 ubiquitination of the non-functional 80S ribosome induces subunit dissociation by Slh1, leading to degradation of the non-functional 18S rRNA (Sugiyama et al. 2019).

Eva Kummer (Nenad Ban Lab) reported how mitochondrial ribosomes are rescued from stalling induced by truncated mRNAs by showing a cryoEM structures of ribosome-bound mitochondrial rescue factor. Kummer showed that the factor senses stalled ribosomes based on the occupancy of the mRNA entry channel and occupies the vacant channel left by a truncated mRNA.

The ribosome-associated protein quality control (RQC) mediates the degradation of nascent polypeptides that remain attached to 60S subunits after ribosome dissociation (Joazeiro 2019). RQC includes the E3 Ligase Ltn1/Listerin for substrate ubiquitination and Rqc2/NEMF. Remarkably, Rqc2 can add C-terminal alanine and threonine residues (CAT-tails) to the arrested nascent chain in a template-free manner. This increases efficiency of ubiquitination by Ltn1 but can also cause aggregation of degradation-resistant CATylated proteins, eventually representing a fail-safe mechanism. Onn Brandmann described a novel function of CAT-tails, which can serve as “off-ribosome” degrons, allowing for the degradation of CATylated proteins independent of Ltn1 and their localization at the 60S ribosomal subunit (Sitron and Brandman 2019). He further showed that CATylation becomes important for growth of yeast cells when stressing cell by cycloheximide addition in absence of Ltn1.

Roland Beckmann presented cryoEM structures of Vms1 in complex with a 60S ribosomal subunit. Vms1 is part of the RQC system and cleaves the peptidyl-tRNA to release the stalled nascent polypeptide chain (Joazeiro 2019). Beckmann reported on two structures, representing pre- and post-hydrolysis tRNA cleavage states (Su et al. 2019). Vms1 binds via its release-factor domain in the ribosomal A-site. The bound peptidyl-tRNA is in an intermediate state between the A/P sites before cleavage, inducing a local structural change in the tRNA and exposing the Vms1 cutting site in the acceptor arm. This structural state is stabilized by binding of the ATPase Arb1 to the ribosomal E-site. Vms1 binding sterically clashes with Rqc2 and eventually displaces Rqc2 from the 60S subunit, thereby terminating CATylation and releasing the CATylated polypeptide from the ribosome.

## Regulation of stress responses

Eilika Weber-Ban reported on a novel pathway for the DNA damage response in Mycobacteria. Central to this pathway are the two proteins PafB and PafC that are encoded in an operon with PafA, which mediates the ligation of the prokaryotic ubiquitin-like protein Pup to substrate proteins to target them for degradation. PafB and PafC contain winged helix-turn-helix DNA-binding motifs and deletion of the PafB or PafC encoding genes renders *Mycobacterium smegmatis* susceptible to DNA stress. PafB and PafC interact with each other and activate some 150 genes involved in DNA recombination and repair, whereas the SOS regulon, the classical DNA damage pathway in *E. coli*, only comprised 25 genes in *Mycobacteria*. Furthermore, PafBC indirectly also influences the SOS response, since RecA, the DNA damage sensor and co-activator of the SOS response, is among the PafBC targets. Thus, PafBC is the major regulator of DNA repair and maintenance in mycobacteria (Muller et al. 2018). Interestingly, 26 of the 152 PafBC targets are pupylated and the pupylation system degrades RecA during recovery from DNA stress, indicating that positive and negative regulators of this pathway are encoded within the same operon.

Matthias P. Mayer reported on two conceptionally similar central regulators of stress, p53 and Hsf1. Both transcription factors are oligomeric proteins (p53 tetramer, Hsf1 trimer) and integrate a large number of stress signals from DNA and proliferation (p53) and protein homeostasis (Hsf1). Both transcription factors are regulated by the Hsp70 system which interferes with DNA binding and transcriptional activation. However, the molecular mechanism by which Hsp70 achieves this regulation is different. In p53, Hsp70 in cooperation with its J-domain co-chaperone DnaJB1/Hdj1 binds to the DNA-binding domain in a DNA competitive manner and locally unfolds the DNA-binding domain to convert it into a conformation found in many cancer-related mutant p53 proteins (Boysen et al. 2019). In contrast, dissociation of Hsf1 from DNA is achieved by monomerization using an entropic pulling mechanism.

Whether such a mechanism of Hsf1 regulation is also operative in yeast where Hsf1 is constitutively trimeric and believed to be permanently DNA bound was discussed by Claes Andréasson. He managed to isolate yeast Hsf1 in an ATP sensitive complex with the yeast Hsp70 Ssa1 and the J-domain co-chaperone Sis1 recombinantly expressed in *E. coli*. His data suggest that Hsp70 negatively regulated Hsf1 by preventing DNA binding, consistent with former results (Zheng et al. 2016). Overexpression of the Sse1 nucleotide exchange factors in the nucleus induces the heat shock response, indicating that it is the nuclear pool of Hsp70 that maintains Hsf1 in a latent state. Accordingly, accelerating or freezing the Hsp70 ATPase cycle by nuclear overexpression and deletion of the nucleotide exchange factor FES1 induces a strong heat shock response.

Lea Sistonen next reported on transcriptional memory of RNA Polymerase II pausing. She shows that a single heat shock can prime cells to react to a subsequent heat pulse with accelerated Polymerase II transcription in a large number of genes and faster repression in other genes and that such a memory can be maintained over mitotic divisions. Repeated stress exposure changes basal and inducible transcription increasing for example the transcription of the HSPA8 gene, and alters the distribution of transcribing RNA Polymerase II complexes at specific regions in 3′UTRs. Decelerated transcription termination co-occurs with reduced transcript cleavage. Transcription is not only altered at the promoter but also at enhancer sites and Hsf1 is indispensable for heat-induced gene and enhancer activation in unconditioned and preconditioned K562 cells. Hsf1 action can also be mediated through enhancer transcription.

The heat shock response in the tumor microenvironment was the focus of the presentation by Ruth Scherz-Shouval. The stromal stress network seems to be essential for the phenotypic plasticity and fitness of tumors. Tumors are highly heterogeneous ecosystems in a stressful environment. Different stress responses and in particular the heat shock response and the ER stress response are activated in cancer cells as well as cells of the tumor microenvironment, to promote tumor fitness via cell autonomous and non-cell-autonomous mechanisms. There is cross talk between these stress responses in the tumor microenvironment, in particular in cancer-associated fibroblasts (CAFs). Hsf1 is activated upon inflammation and promotes a wound-healing response. During chronic stress, this response promotes tumor growth. Hsf1 is activated in cancer-associated fibroblasts (CAFs) and high stromal Hsf1 activity is associated with poor prognosis. Hsf1 activity in CAFs seems to be required for the deposition of extracellular matrix (ECM) and loss of Hsf1 affects ECM remodeling in colon cancer.

Another network important for cancer progression was analyzed by Ritwick Sawarkar. As high nuclear concentrations of Hsp90 correlate with poor prognosis, Hsp90 seems to play an important pro-tumorigenic role in the nucleus. In order to identify a regulatory module that is chaperoned by Hsp90, a number of complementary screens for synthetic genetic interactions were performed, including knockdown screens for synthetic lethality with or more resistant to Hsp90 inhibition. A large number of genes were shown to have synthetic interactions with Hsp90 inhibition. About half of them showed nucleus-specific interaction with Hsp90 and many of them are co-expressed in 17 different cancer types. Among the identified proteins are several components of chromatin remodeling complexes, in particular one that controls expression of cell cycle genes. Inhibition of this complex compromises cell survival in synergism with Hsp90 inhibition.

Anne Bertolotti first showed an update on her work on the drug Guanabenz (GB) that protects cells from ER-UPR-inducing proteotoxic conditions. GB transiently inhibits the regulatory subunit R15A of the PP1C phosphatase that dephosphorylates eIF2α, thus prolonging stress-activated translational inhibition. A derivative of this GB that has no alpha-2 adrenergic side effects and is CNS permeable has shown efficacy in disease models in mice where ER stress is activated. The idea of specific targeting of the regulatory subunits of PP1-phosphatase complexes, especially for disease-causing proteins that do not act on the ER-UPR, was further explored after the identification of raphin-1 as a specific binder to R15B. This binding causes R15B degradation and results in a transient inhibition of constitutive translation with effectiveness in a mouse model for mutant Huntington’s disease (Krzyzosiak et al. 2018). Anne emphasized the importance of the transient effects of both drugs as key to their tolerability. The idea is that effectiveness is caused by an increase in availability of chaperones that otherwise are engaged in co-translational folding.

Many different forms of stress, including ER stress, viral infections, oxidative stress, acute heme-depletion, and metabolic stress, induce the so-called integrated stress response by activating stress-specific protein kinases (PERK, PKR, HRI, GCN2) to phosphorylate eIF2 and thus down-regulate translation initiation. In a shared talk, Aditya Anand (Peter Walter Lab) reported on ISRIB, a drug-like small-molecule activator of the eIF2B nucleotide exchange factor. eIF2B activity becomes limiting in stressed cells as it is tied up in an inactive complex after tightly binding to eIF2•P. ISRIB replenishes the depleted pool of eIF2B by promoting assembly of more decameric eIF2B from two hetero-tetrameric and one homodimeric building blocks. Anand reported on the cryoEM structure of the eIF2B decamer in complex with ISRIB (Tsai et al. 2018) and on the structure of the eIF2B decamer in complex with eIF2 and eIF2•P (Kenner et al. 2019). It turned out the phosphorylation of eIF2 alters the structure of the so-called S-loop of eIF2’s alpha subunit, allowing eIF2·P to bind to a specific surface of the eIF2B decamer, thus converting it from a substrate to an inhibitor of eIF2B. These findings can explain why ISRIB blocks the integrated stress response only when eIF2·P is limiting, resulting presumably in a bell-shaped activity window, and why ISRIB shows no overt toxicity in rodent models (Rabouw et al. 2019). The assembly of eIF2B emerges as a rheostat for translation inhibition. Because ISRIB enhances cognition, in particular after traumatic brain injury, these results promise to be of high clinical relevance.

Peter Walter reported on the focus formation of IRE1 in response to ER stress. It is intriguing that the Hill coefficient for the RNase activity of IRE1 is 3.4 suggesting foci-formation to be responsible for a high cooperativity of IRE1. Fluorescence recovery after bleaching (FRAP) experiments demonstrated a rapid but only partial recovery of fluorescence, suggesting that a core of IRE1 has limited mobility. Correlative light-electron microscopy and cryoEM tomography with subtomogram averaging revealed lumenal filaments in a well-ordered double helical arrangement, presumably composed of IRE1, in regions of a highly curved ER membrane network built of narrow, convoluted tubes of 30 nm diameter lacking attached ribosomes. The large surface/volume ratio of these structures may shape IRE1’s signaling response.

## Protein phase separation as novel proteostasis strategy

The formation of membrane-less, phase-separated compartments has recently attracted major attention and is subject of intense research. The sequestration of cellular components during adverse growth conditions allows for regulation of their activity states and temporal storage. The formation of phase-separated assemblies invokes cellular factors that typically harbor intrinsically disordered segments, which are crucial for sequestration activity (Franzmann and Alberti 2019).

Simon Alberti reported on the mechanism of stress granule (SG) formation in human cells. SGs are largely composed of mRNA- and RNA-binding proteins and form during a multiplicity of stress conditions that inhibit protein translation. SG formation critically depends on the RNA-binding proteins G3BP1/2, which harbor a disordered RGG domain for RNA binding and an acidic region. Alberti showed that purified G3BP1/2 specifically binds to unstructured, naked mRNAs that is typically released from disassembling polysomes during stress, and these mRNAs are then sequestered in liquid droplets formed by G3BP1/2-driven phase separation. G3BP1/2 activity is controlled by its acidic region, which inhibits the RGG domain by causing formation of a closed, inactive structure. Unstructured mRNAs released during stress from polysomes compete with the acidic domain for binding to the RGG domain, thus causing an opening of the G3BP1/2 structure and enabling mRNA sequestration. This initiation phase of SG formation is followed by partitioning of other SG marker proteins (e.g., TIA, FUS) into G3BP1/2 droplets, leading to the formation of mature SGs.

Manajit Hayer-Hartl reported on the formation of carboxysomes, which represent a specific phase-separated microcompartment of cyanobacteria. Carboxysomes contain high concentrations of the carbon fixing enzyme Rubisco that is composed of eight large (RbcL) and small (RbcS) subunits. The protein CcmM is required for β-carboxysome formation and interacts with Rubisco. Hayer-Hartl showed that CcmM sequesters Rubisco into dynamic liquid droplet-like structures (Wang et al. 2019). CcmM activity relies on its three SSUL (Rubisco small-subunit like) domains and is modified by disulfide bond formation within some SSUL domains, controlling compartment dynamics. A cryoEM structure of the CcmM SSUL domains in complex with Rubisco showed that only one out of the three SSUL domains binds to a peripheral site of the holoenzyme, allowing the remaining SSSL domains to create a meshwork of CcmM/Rubisco complexes (Wang et al. 2019).

Elena Hidalgo reported on the formation of “protein aggregate centers” (PACs) in *Schizosaccharomaces pombe* (fission yeast) that form under heat stress conditions and are composed of misfolded proteins. PACs colocalize with Hsp40, Hsp70, and small heat shock (sHsp) chaperones, suggesting they could function as protein quality control centers. Hidalgo showed that the Mas5-Ssa2 (Hsp40-Hsp70) chaperones are essential for PAC formation. Loss of ribosome-bound Hsp70 (RAC) or the disaggregase Hsp104 also reduces PAC formation. PACs are suggested to protect sequestered proteins from proteasomal degradation, allowing for their reflux into the cellular proteome upon stress relief.

Serena Carra reported how cells handle the accumulation of defective ribosomal products (DRIPs). She showed that DRIPs accumulate in specific subcompartments of nucleoli, including nucleolar aggresomes and amyloid-bodies, which are the same structure. DRIPs are only transiently stored and cleared in an Hsp70-dependent manner upon stress relief. Upon stress, DRIPs are sequestered at nuclear PML bodies and are cleared by Hsp70 and the AAA+ dislocase p97/VCP. These findings qualify PML bodies and nucleoli as overflow compartments to protect cells from aberrant polypeptides. She also showed that there is a trade-off between nuclear proteostasis and genome integrity: upon proteotoxic stress, cells use ubiquitin mainly to label misfolded proteins for degradation that accumulate in these nuclear quality control compartments, to the detriment of ubiquitin-dependent DNA repair (Mediani et al. 2019).

Small heat shock proteins participate in the sequestration of misfolded proteins. They are composed of a conserved α-crystallin domain and variable, disordered N- and C-terminal extensions that are important for substrate interactions and sHsp oligomerization. Hartmut Oschkinat reported on the structural analysis of the human sHsp αB-crystallin by solid state NMR. αB-crystallin forms polydisperse oligomers, which are composed of dimeric building blocks formed by α-crystallin domains. Higher oligomer formation is driven by N- and C-terminal extension harboring a crucial IxI/V motif. Oschkinat showed that half of the C-terminal IxI motifs are complexed within the oligomer, whereas half stay unbound, contributing to plasticity and heterogeneity of sHsp complexes.

## Amyloid formation, protection, and degradation

Frederic Rousseau showed that most, if not all globular proteins, are enriched in amyloidogenic protein sequences of 7–10 amino acids that are not only unique and thus can be used as a barcode in e.g. protein detection in westerns, but that also can be targeted with peptides to specifically aggregate any protein of interest and inactivate the function of the protein. This was exemplified by targeting the amyloidogenic protein sequences in VEGFR2 which resulted in amyloid-related toxicity only in cells that express and depend on VEGFR2 activity (Gallardo et al. 2016). Similarly, such peptides can be used for anti-microbial and anti-viral purposes.

Paola Picotti reported on her efforts to understand protein aggregation in a cellular context. Limited proteolysis coupled to mass spectrometry (LiP-MS) was used for in situ analysis of protein structural changes through detection of their proteolytic fingerprints. The method allows identifying structurally altered proteins upon cellular perturbations on a proteome-wide scale and directly from complex biological extracts, and pinpointing altered regions with a resolution of around 10 amino acids. This LiP-MS method was applied to track conformational transitions of α-synuclein. It was possible to distinguish unfolded, helical, oligomeric, and amyloid forms of α-synuclein using their characteristic proteolytic signatures. The LiP-MS method was also applied to detect structurally altered proteins in the cerebrospinal fluids of Parkinson’s disease patients compared with healthy control individuals that show potential as structural biomarkers. Another application of the LiP-MS method was the identification of interactions of proteins with small molecules. Small molecules such as drugs or metabolites are added to cell extracts and their protein targets are detected based on alterations of their proteolytic patterns at the binding sites. Within 40 cases of interactions with known targets, in 36 cases, the detected peptides were less than 4 Å away from the binding site. Using 20 endogenous metabolites, the Picotti group found 1678 metabolite-protein interactions, 1447 of which were novel and 231 known sites. These findings suggest that metabolites may have a broad range of functions and enzymes may be more promiscuous than previously assumed. The approach is currently applied to discover mechanisms of action and binding sites of anti-amyloidogenic compounds.

Helen Saibil reported on the ultrastructure of amyloid fibers generated by expression of the exon-1 fragment of polyQ-hungtintin in HEK293 cells. The amyloid fibers are highly immobile (solid) and can arise by conversion of initially formed liquid phase–separated droplets. Indeed in vitro data on mutant huntingtin shows that fiber bundles grow out of liquid like-dots. Both in vitro and in yeast cells, the liquid droplets are reversed by hexanediol, but traces of fibrils remain, which can nucleate new droplets after removal of the hexanediol (Peskett et al. 2018). She also reported on collaborative work with the Bukau group showing that α-synuclein fibers are decorated with DNAJB1, HSP70, and Apg-2 (Hsp110) that can fragment these fibers into products that are less toxic when added extracellularly to cells (Gao et al. 2015).

Anne Wentink (Bernd Bukau Lab) reported on the molecular mechanism of α-synuclein amyloid fibril disaggregation by Hsp70, DnaJB1, and Apg2. Using solution NMR, she identified the binding sites of Hsp70 and DnaJB1 in α-synuclein required for disaggregation by the Hsp70 system. She demonstrated that DnaJB1 provides specificity for the oligomeric state and mediates clustering of Hsp70s at the fibril surface, which is required for activity. Efficient disaggregation requires also the nucleotide exchange factor Apg2. The alternative Bag1 nucleotide exchange factor, though efficient in catalyzing nucleotide release, supports α-synuclein disaggregation poorly. The reason seems to be the difference in molecular weight, as increase of the size of Bag1 enhances its support for α-synuclein disaggregation. This implies that the Hsp70 system relies on the unique properties of specific co-chaperones for fiber disassembly.

Margreet Koopman (Stefan Rüdiger Lab) reported on the disaggregation of TAU fibrils by the human Hsp70 chaperone machinery. Negative stain EM reveals that Alzheimer’s disease–related TAU fibrils come in two flavors, paired helical filaments and straight filaments. Hsp70-binding sites are found at the end of the aggregation-prone core. Using sucrose gradient centrifugation and single-particle tracking via light scattering, she demonstrated that the number of bigger particles decreases and the monomers appear upon incubation with the Hsp70 system. Thereby, she found that Hsp70 acts differently on paired helical filaments than straight filaments. The conformation of the filaments may determine the mode of interaction.

Janine Kirstein, using a novel FRET-based assay for polyQ aggregation (Scior et al. 2018), addressed which chaperones are rate-limiting in the prevention or polyQ aggregation. A combination of Hsp70, Apg2, and DNAJB1 completely suppressed aggregation. Also, in *C. elegans*, class B J-domain proteins were among the strongest suppressors. Especially DNJ-13 (the closest DNAJB1 ortholog) strongly suppressed together with HSP-1 and HSP-110 (Hsc70 and Apg2 orthologs) the aggregation and was found to bind with its C-terminus to the poly-proline region of the huntingtin that resides C-terminal of the polyglutamine stretch. As such, its action differs from TRiC that suppresses huntingtin aggregation by binding to the region N-terminal of the polyQ region and from DNAJB6 and NAC that suppress huntingtin aggregation and directly bind to the polyQ stretch.

Tejbir Kandola also reported on a novel in vivo cytometric technique, Distributed Amphifluoric FRET (DAmFRET) to detect and quantify the concentration-dependence of various protein self-assemblies with high sensitivity and single-cell resolution (Khan et al. 2018). This technique was shown to work on several types of aggregate-forming proteins as well as confirm the behavior of assembly-modulating mutants. It allows for high-throughput analysis of critical concentrations for soluble proteins to convert to aggregates and how chaperones affect such conversions (Venkatesan et al. 2019).

Ursula Jakob showed her progress on the chaperone-like activities of polyphosphate (PolyP). PolyP drastically changes the structure of amyloid fibers (e.g., α-synuclein). It binds to α-synuclein after amyloid fibers are formed and does so via electrostatic interactions. Hereby the resulting fibers have reduced toxicity when added to cells. Also, the uptake of those fibers is reduced by the action of polyP. In the brain, polyP is clearly present in neurons. Inside cells, polyP levels are high especially within the nucleolus, a compartment suggested to be important for nuclear protein quality control.

## Quality control and UPS

Michael Rape reported on the molecular mechanism of their recently discovered dimerization quality control (DQC) pathway, which can selectively bind and degrade inactive heterodimers of BTB domain containing proteins, while leaving active homodimers intact (Mena et al. 2018). Surprisingly, X-ray analyses showed that heterodimers are structurally indistinct from their homodimeric counterparts. Instead of detecting protein misfolding, evidence was provided to show that the core E3 ligase of the DQC pathway, SCF^FBXL17^, ultimately captures a monomeric BTB domain and therefore tests complex stability. Heterodimers do not form the interdomain connections via N-terminal beta-strands as they do in homodimers, which facilitates dimer dissociation, recognition by the DQC pathway, and degradation.

Sebastian Schuck reported on the stress-induced, homeostatically regulated protein degradation (SHRED) pathway that he recently discovered using a reporter for quality control at the ER membrane (Szoradi et al. 2018). This pathway relies on the proteins Roq1, Ynm3, and Ubr1 for degradation of misfolded proteins. Roq1 is induced by various stresses and is cleaved by Ynm3 at a specific position, exposing an arginine at the new N-terminus and thereby becomes a substrate of the N-end rule ubiquitin ligase Ubr1. Once cleaved, Roq1 is bound and Ubr1 is reprogrammed to ubiquitinate misfolded proteins and target them for degradation. This pathway is also induced by starvation. Using a fluorescent timer-based screen, he identified natural SHRED substrates including the translation elongation factor Tef4, suggesting that SHRED promotes downregulation of translation during starvation.

Thorsten Hoppe showed his results of a genetic screen for microRNAs that affect the stability of the proteasomal ubiquitin-fusion degradation (UFD) substrate UbV-GFP and focused on the role of MIR-71 that was reported to be relevant to stress resistance and longevity in *C. elegans* (Boulias and Horvitz 2012). In addition to the UFD pathway, worms lacking MIR-71 also showed defects in ER-associated degradation (ERAD) downstream of substrate ubiquitination. The impact of MIR-71 in proteostasis is based on its regulation of *tir-1* mRNA level, encoding a protein involved in innate immunity and neuronal activity, TIR-1, that is specifically expressed in a stress-regulated, MIR-71 dependent manner in olfactory neurons. The TIR-1/MIR-71 axis is the key to the cell non-autonomous regulation of protein homeostasis in the gut and linked to the olfactory food response in worms. It was speculated how a similar connection may exist in humans provoked by the findings that patients with Parkinson’s disease display both smelling defects and obstipation.

Jason Gestwicki reported on presumably chaperone-independent functions of the Hsp70/Hsp90 co-chaperone CHIP (Ravalin et al. 2019). The tetratricopeptide repeat (TPR) domain of the E3 ubiquitin ligase, CHIP, is known to bind the conserved EEVD motif at the C-terminus of nuclear-cytosolic Hsp70s and Hsp90s, targeting an E2 enzyme to chaperone clients for ubiquitination and proteasomal degradation. To better understand molecular recognition in this system, the Gestwicki group first determined the absolute specificity of CHIP’s TPR domain using a peptide library approach. Surprisingly, they found that the EEVD motif is not the optimal CHIP-binding sequence. Based on these screening results, they identified few human ORFs having a predicted CHIP-binding site at their C-terminus. However, in the DegraBase (Crawford et al. 2013) they found approximately 2700 cryptic CHIP-binding sites that are only made accessible through caspase cleavage. One of these proteins is the microtubule-associated protein TAU. Indeed, in validation studies, they found that caspase-cleaved TAU, but not full-length TAU, binds to CHIP, which then rapidly ubiquitinates TAU in vitro. Interestingly, another protein on the list is caspase-6, which is an enzyme that cleaves TAU at the cryptic CHIP-binding site. Indeed, self-cleavage of caspase-6 exposes a CHIP-binding site and this interaction allosterically suppresses the activity of caspase-6. Based on these results, Gestwicki proposed an integrated model for TAU homeostasis, in which CHIP suppresses cleavage of TAU, while also removing cleaved TAU by ubiquitination. Consistent with this model, they found that neuronal CHIP levels decrease in Alzheimer’s disease (AD). Thus, integration of caspase activity and CHIP recognition might be important for homeostasis of TAU and possibly other proteins.

## Quality control in the ER

The cystic fibrosis transmembrane conductance regulator (CFTR) folds co-translationally in and at the ER into 5 separate domains, initiated by rapid folding of the nucleotide binding domain 1 (NBD1) onto which the two transmembrane domains assemble next, followed by the second NBD (NBD-2). NBD1 folding is impaired in the so-called deltaF508 mutant, the most common mutation causing Cystic Fibrosis (CF), which leads to its accelerated degradation. Wild-type CFTR is protected from degradation in a Hsp90- and Hsp70-dependent manner. Ineke Braakman reported on a collaboration with Georgios Karras (Houston TX): they probed 60 CF-causing mutants (covering > 96% of all CF patients) and showed how most of them indeed bind more Hsp90 (and Hsp70). Especially mutations in the NBD1 domain are hypersensitive to limited proteolysis (van Willigen et al. 2019) and are bound more by chaperone. This implies that reduced stability, increased chaperone recognition, and accelerated degradation are common characteristics in many CF mutants, which suggest that manipulating chaperone-CF affinity (e.g., by knockdown of co-chaperones like Aha-1 (Wang et al. 2006) may have therapeutic potential in CF patients.

Kazuhiro Nagata reported on novel findings related to the ER-resident J-domain protein ERdj5. Previous work in his group had already shown that ERdj5 is a BiP-co-chaperone with reductase activity that plays a role in ERAD and also regulates calcium-homeostasis inside the ER (Ushioda et al. 2016). However, it was unclear how ERdj5 would obtain reductase activity. Here, Nagata showed the electron transfer from nascent chains to ERdj5 through Ero1-PDI complex. These findings revealed a novel electron transfer pathway in the ER lumen. Then, he showed data on the translational regulation of ERdj5 and showed how it pauses translation. This pausing is released during ER stress through BiP interaction. It suggests that BiP helps to pull out ERdj1 from the ribosome and regulates the amount of the reductase in the ER.

Michael van der Weijer (Carvalho Lab) used a CRISPR/Cas9-based screen to delineate a novel ERAD complex responsible for the degradation of distinct transmembrane proteins. The data revealed that there is a high level of complexity and specificity of different E3 ligases targeting distinct transmembrane proteins for ERAD.

## Protein homeostasis, aging, and age-related disease

Many data have shown that protein homeostasis declines with aging. In *C. elegans*, this seems to be a programmed and, in part, non-cell autonomous process as summarized by Rick Morimoto (Labbadia and Morimoto 2015a). The stress-induced response (heat shock response and unfolded protein response) declines in response to signals from mature germ line stem cells. This results in transcellular serotonin signaling that causes chromatin condensation throughout the organism, e.g., preventing HSF-1 binding to target genes (Labbadia and Morimoto 2015b; Tatum et al. 2015). Interestingly, in egg cell mutants, this shut-down is not seen and longevity is increased, supporting the tight connection between reproductive capacity and stress resilience.

Anat Ben-Zvi next showed that while this gonadal pathway is particularly important under acute stress responses (Shemesh et al. 2013), another longevity pathway activated by dietary restriction (*eat-2*) seems to particularly act during chronic stress like the expression of aggregation-prone proteins. This pathway can be activated later in life, and is functional in proteostasis maintenance (Shpigel et al. 2019) despite this inhibitory chromatin state.

Patricija van Oosten-Hawle continued on the theme of cell non-autonomous regulation of protein homeostasis by showing how glutamatergic neuronal signaling can induce Hsp90 chaperone expression to suppress amyloid-beta aggregation and toxicity in distant tissues. This effect requires the transcription factor PQM-1 of which the activation is prolonged by Hsp90. Among PQM-1-activated genes, the innate immunity-associated protein CLEC-41, which is predicted to interact with ionotropic glutamate receptors, was found to be essential for this neuronal signaling (O'Brien et al. 2018).

Also, in human primary fibroblasts, age-related senescence is associated with a collapse of protein homeostasis. Reut Shalgi showed that, whereas basal chaperone levels are largely unaffected, senescent fibroblast shows reduced ability to activate stress responses (HSR and UPR), but also has an impaired control over stress-related control of splicing. So, aging is associated with a general failure to induce programs that respond to stress situations.

Protein aggregation not only hallmarks many age-related degenerative diseases, but the process of protein aggregation should be considered also as a driver of these diseases according to Harm Kampinga. He argued this on the basis of findings that in all cases, genetic forms of the disease affect either protein stability of the disease-related protein or affect protein quality control. In fact, many diseases caused by mutations in chaperones (chaperonopathies) are also aggregation pathologies. He exemplified how both dominant negative effects of chaperone mutants in Bag3 (Meister-Broekema et al. 2018) or HSPB5 as well as loss-of-function mutations (in DNAJB6) lead to a collapse in protein homeostasis that causes toxic aggregation. He also provided proof-of-concept data for pharmacologically interference with these dominant negative effects as a means to alleviate such toxicity.

More and more data suggest that certain proteins also can drive other (substrate) proteins into phase separations and that such activity can accelerate aggregation of the substrate proteins. Deletion of such proteins (ceMOAG-4/hSERF-2) was previously shown to suppress the formation and toxicity of polyglutamine aggregation (van Ham et al. 2010). Based on an extensive screen in collaboration with the group in Leuven (Rousseau and Schymkowitz), Anita Pras (Nollen Lab) showed how ceMOAG-4/hSERF-2 can act on many structurally unrelated aggregation-prone substrates via binding to negatively charges regions in these substrates via its positively charged N-terminal region, making ceMOAG-4/hSERF-2 an attractive generic target for inhibition in multiple protein aggregation diseases.

A disease that is clearly at the cross-road of functional phase separation and aggregation pathologies is amyotrophic lateral sclerosis (ALS). A central feature of all forms of genetic and non-genetic ALS is the aggregation of TDP-43. Magdalini Polymenidou reported on her recent findings related to both the physiological and pathological states of TDP-43 isolated from cellular and animal ALS models and patient samples using a newly developed method called SarkoSpin (Laferriere et al. 2019) and subsequence analyses by mass spectrometry. These analyses revealed that TDP-43 forms stable assemblies with distinct morphologies, differential neurotoxicity, and different seeding capacity and disease duration. Hence, different TDP-43 conformations may be a key to the large disease heterogeneity seen in ALS.

There is increasing awareness that inflammation is not only associated with protein aggregation disease, but also that aggregates may actually be a major trigger for (neurotoxic) immune responses. Cintia Roodveldt showed how exogeneous TDP43 aggregates can be rapidly internalized by microglia to cause a dysregulated immune response (Leal-Lasarte et al. 2017). The key to this reaction was the binding of the internalized aggregates to MAPK/MAK/MRK overlapping kinase (MOK) that drives key signaling pathways related to neuroinflammation.

Finally, Anna Ponomarenko addressed an entirely different disease area, i.e., the infection which RNA viruses, which remain a pandemic threat. Specifically, influenza virus possesses high mutation rates, which enable effective immune system escape. However, rapid mutations accumulation imposes biophysical challenges on proteins. The complexity of viral proteome folding and necessity of the host chaperonome assistance suggested the hypothesis that depletion of the host chaperones can reveal the biophysical defects of mutant viral proteins (Phillips et al. 2018). Proof-of-concept data were provided showing that downregulation of heat shock factor 1-controlled chaperones renders immune escape viral protein variants destabilized and unable to propagate and thus restricting viral replication.
